# The Role of Disgust in Male Sexual Decision-Making

**DOI:** 10.3389/fpsyg.2018.02602

**Published:** 2019-01-22

**Authors:** Megan Oaten, Richard J. Stevenson, Caley Tapp, Trevor I. Case, Allie Cousins

**Affiliations:** ^1^School of Applied Psychology, Griffith University, Gold Coast, QLD, Australia; ^2^Department of Psychology, Macquarie University, Sydney, NSW, Australia

**Keywords:** disgust, disease, sexual arousal, contamination, decision making

## Abstract

Sexual arousal is known to increase risky behaviors, such as having unprotected sex. This may in part relate to the emotion of disgust, which normally serves a disease avoidant function, and is suppressed by sexual arousal. In this report we examine disgust's role in sexual decision-making. Male participants received two study packets that were to be completed at home across two different time-points. Participants were asked to complete one packet in a sexually aroused state and the other in a non-aroused state. Participants were asked to rate: (1) arousal, (2) disgust, (3) willingness for sex, and (4) disease risk toward a range of female targets, which varied in level of potential disease risk (sex-worker vs. non sex-worker) and attractiveness. A measure of trait disgust was also included along with other related scales. Sexual arousal was associated with reduced disgust and reduced judgments of disease risk for all targets—these latter two variables being correlated—and with enhanced willingness to have sex with all of the depicted persons. Willingness to have sex when aroused (in contrast to non-aroused) was predicted by disease risk judgments and trait disgust, suggesting both direct (state) and indirect (trait) effects of disgust on sexual decision-making.

## Introduction

Sexually transmitted infections (STI) are common, with around 0.5 billion new cases occurring each year (World Health Organization, 2013). These are associated with considerable morbidity and mortality, notably from HIV infection, fetal syphilis and cervical cancer (World Health Organization, 2013). Much of the STI disease burden falls on young people, who while accounting for around a quarter of the sexually active population, contract around half of all diagnosed STIs (Eaton et al., 2008; Centers for Disease Control, 2012). Given these statistics, an important consideration is the process by which young people make sexual decisions as they relate to STI risk. In this report we examine the role of the emotion of disgust in this process. As we describe below, disgust seems to be involved in driving avoidance of disease transmitting objects (e.g., body products; Curtis et al., 2004; Oaten et al., 2009) and people (e.g., Ryan et al., 2012), and it has been suggested that it may perform a similar role in the sexual domain (e.g., Stevenson et al., 2011; Borg and de Jong, 2012). We chose to only include young men because our previous work indicated that disgust toward sex-related cues could be reduced in male participants during heightened sexual arousal, and we were interested to test whether this effect could also be observed in sex-related disease avoidance behavior (e.g., Stevenson et al., 2011).

Two important variables have been identified that affect sexual decision-making as they pertain to STI risk perception. The first, and most potent, is sexual arousal. Blanton and Gerrard (1997), appear to have been the first to demonstrate in male participants that judgments of STI risk were significantly reduced by sexual arousal. Several subsequent studies have tended to confirm this result, finding first, that sexual and non-sexual risk taking in both men and women is enhanced by sexual arousal (Skakoon-Sparling et al., 2016) and second, that feelings of self-control and sexual-restraint are reduced (Skakoon-Sparling and Cramer, 2016). Although, one study reports that sexual arousal only affects risk taking as it pertains to sexual behavior (Imhoff and Schmidt, 2014), the broad and well-supported finding is that enhanced sexual arousal alters behavioral intentions, such that activities that might be deemed risky and unacceptable when unaroused are deemed more acceptable and less risky when aroused (Ariely and Loewenstein, 2006).

A second and related factor is physical attractiveness. Two studies have explored the impact of this variable on sexual decision-making. In one report HIV+ gay men were asked to make judgments about a series of scenarios when in a sexually aroused and in a non-aroused state (Shuper and Fisher, 2008). Intention to have unprotected sex was independently reduced by the attractiveness of the potential partner, the potential partner's HIV status and sexual arousal. Similar findings have also been obtained in heterosexual men and women, with increased willingness to have unprotected sex with an attractive partner, relative to a less attractive one (Epstein et al., 2007). While these two studies did not directly ask for STI risk judgments, it is apparent that the behavioral intentions carry a heightened risk of disease transmission, and that this heightened risk is very likely to be known to the participant.

If a state of sexual arousal and an attractive potential partner increase the likelihood of making a suboptimal sexual decision—e.g., having unprotected sex—an obvious question is why? One possibility, and the focus of this study, is the emotion of disgust. Apart from the broader observation made earlier that disgust serves a disease avoidance function (e.g., Curtis and Biran, 2001; Marzillier and Davey, 2004; Oaten et al., 2009; Fleischmann and Fessler, 2011), three lines of evidence suggest it may be a significant contributor to STI-related sexual decision-making. First, Stevenson et al. (2011), examined the effects of sexual arousal on male participants' disgust reactions to sex-relevant and sex-non-relevant disgust elicitors, in the visual, auditory and tactile modalities. Sexual arousal selectively reduced disgust for sex-relevant elicitors, but not for sex-non-relevant elicitors. Similar findings were also obtained by Borg and de Jong (2012), but with female participants. A further study examined whether sexual arousal would affect a self-report measure of disgust in men and women, and found evidence of reduced disgust for sex-related questions, but solely in women (Lee et al., 2014). Although there is some uncertainty about the nature of disgust in non-human species, it has been suggested that similar decision processes may also occur here, with sexual arousal suppressing the normal avoidance of diseased, but sexually available, conspecifics (e.g., brief exposure to female odors enhanced the willingness of male mice to approach infected females; Kavaliers and Choleris, 2013).

A second line of evidence that points to disgust's involvement in sexual decision making is the finding that eliciting a state of disgust can inhibit sexual arousal in both men and women. Priming disgust in participants leads to reduced arousal-related judgments of erotica (Andrews et al., 2015), with this effect also obtained in a further study using just female participants (Fleischman et al., 2015). Relatedly, it has also been suggested that elevated trait disgust sensitivity may be a contributory factor to sexual dysfunction in women, by inhibiting sexual arousal (e.g., Van Overveld et al., 2012). A third line of evidence comes from finding that lower trait based measures of disgust, especially that pertinent to sex-related elicitors, are associated with a greater number of short-term sexual partners in heterosexual men and women (Al-Shawaf et al., 2015), and in gay men (Zhang et al., 2017). In sum, the general suggestion here is that disgust normally inhibits disease-related contact and that sexual arousal suppresses this emotionally driven avoidance—presumably to aid the greater goal of procreation (de Jong et al., 2013).

In the current study we aimed to examine the role of disgust—both *in reaction* to the stimuli in the study (i.e., state) and as a trait (i.e., disgust sensitivity)—in sexual decision-making, as it relates to STI risk. To examine this, we asked young men to make four types of judgment about a range of potential female sexual partners: (1) how disgusting they found the potential partner, (2) how likely they were to contract an STI, (3) how arousing they found the potential partner, and (4) their willingness to have sex with them. The disgust rating formed our stated-based judgment of this emotion. STI risk rating allowed us an over-arching assessment of disease related knowledge, irrespective of what factors might contribute to it. Ratings of arousal and willingness to have sex were presumed to assess different aspects of behavioral intention—its more distal (i.e., arousal driving approach) and proximal (i.e., consummatory intent) aspects.

Using a fully within-subject design, these judgments were made under two conditions, either when sexually aroused or when in a neutral non-aroused state. In both states participants were asked to evaluate the same set of images (each with a label) depicting four types of potential female sexual partner; attractive sex-workers, unattractive sex-workers, attractive similarly aged controls, and unattractive similarly aged controls. This aspect of the manipulation allowed us to explore two levels of physical attractiveness—a factor known to influence sexual decision making—and two levels of perceived STI risk. Evidence suggests that the prevalence of STIs are higher among female sex-workers than other women (Scott et al., 1995; Mak et al., 2005; Zermiani et al., 2012). This design also allowed us to test interaction effects. Finally, we also obtained a trait measure of core disgust sensitivity (Olatunji et al., 2008), a trait measure of dispositional concerns about disease (Duncan et al., 2009), and the sociosexuality index (Penke and Asendorpf, 2008) to determine attitudes and behavior to short-term sexual relationships.

We hypothesized, first, that heightened sexual arousal would reduce judgments of disgust and disease risk toward all of the depicted female targets—attractive and unattractive, and sex-workers and age-matched controls. Second, we predicted that disgust and disease risk ratings would be positively correlated. Third, we hypothesized that reduced judgments of disgust and disease risk would predict: (1) increases in willingness to have sex and arousal ratings toward all of the images, when contrasting the aroused and unaroused states, (2) reductions in willingness to have sex and arousal ratings toward images of sex-workers, compared to similarly aged controls, and (3) increases in willingness to have sex and arousal ratings toward images of attractive female images vs. unattractive images. Fourth, we hypothesized that state disgust (i.e., measured in the moment) and trait disgust, would exert different effects, reflecting a high or low baseline (i.e., trait), and its exacerbation (i.e., state). However, while we predicted different effects, we were uncertain as to their specific direction, as this was an exploratory and auxiliary hypothesis.

## Method

### Participants

Participants were required to be male and aged 18 years and over. Study packets were initially distributed to 94 eligible participants, with 51 returning data for both the neutral and self-arousal sessions. Community participants were given $20 cash for taking part, with 60-min course credit provided for first-year psychology students. Informed consent was provided by each participant, and the study was approved by Griffith University Human Research Ethics Committee (GU Ref No: 2014/577).

### Design and Study Overview

We used a wholly within-subject design. Participants each completed two sessions at home. In one session they were asked to masturbate to a sub-orgasmic level of arousal and in the other to complete the session in a non-aroused state. On each session they made judgments of arousal, willingness for sex, disgust, and disease risk for a set of images depicting female targets. Two features of the images were manipulated: (1) Whether the female target was attractive or unattractive, and (2) Whether the target was a sex-worker or an age matched control woman.

### Materials

Participants were asked to judge the images of 16 different female targets, with the same images being used in both sessions. These 16 female targets were arranged into four cells: Attractive Sex-worker (ASW), Unattractive Sex-worker (USW), Attractive Age-matched controls (AC), and Unattractive Age-matched controls (UC), resulting in four images per cell. Each image depicts a rear-view of female target—e.g., face not visible to protect the privacy of individual depicted; and each image cues condition (Sex worker vs. Age-matched control) via the clothing and physical stance of the female target depicted. The image dimensions were 8 × 6 cm, and were sourced from Google images (with permissions to use; Image set available via request to author). To definitively categorize the sex-worker or control images, each was paired with one of the following labels: prostitute, escort, sex-worker, hooker, or office worker, secretary, clerical assistant, or book-keeper, and one of the following “attractiveness” vignettes: beautiful, sexy, desirable, attractive, or plain-featured, homely, undesirable, or unattractive, to categorize the attractive or unattractive targets. The images and vignettes had been individually pilot tested on a separate group of participants (*n* = 20) to confirm that the sex-worker and control-related stimuli were classified as such by naïve participants, and that the vignettes corresponded to the attractiveness categories assigned to them. The images were not piloted on attractiveness because female targets were depicted in a rear-view only.

Participants in the current study were required to provide a single rating per image and vignette set, which asked: “How sexually arousing do you find this woman?” (1 = Not at all sexually arousing, 7 = Very sexually arousing); or “Would you be willing to have sex with this woman?” (1 = Not at all willing, 7 = Very willing); or “How disgusting do you find this woman?” (1 = Not at all disgusting, 7 = Very disgusting), or “Are you likely to contract a disease from this woman?” (1 = Not at all likely, 7 = Very likely). The image and vignette combinations were both counterbalanced and randomly allocated (one from each cell) to blocks of four organized by rating category to control for order effects.

Each participant received two experimental packets: a neutral study packet, and an arousal study packet. The neutral study packet contained a participant instruction sheet, demographic questions asking each participant to nominate in an open-ended question format their sex, age, and sexual orientation, and a pre-picture viewing manipulation check which asked participants to rate on a seven-point category scale, “How sexually aroused do you feel right now?” (1 = Not at all aroused, 7 = Very aroused). This was followed by 16 experimental rating sheets each featuring a single female image and vignette (4 from each category—ASW, USW, AC, UC), along with a single rating of either arousal, willingness for sex, disgust, or risk of disease. The final page consisted of a post-picture viewing manipulation check, which asked participants to rate on a seven-point category scale “How sexually aroused do you feel right now?” (1 = Not at all aroused, 7 = Very aroused).

The arousal study packet contained the same materials, albeit with different instructions as detailed further below. In addition, it also contained a small sealable plastic bag and gauze pad to obtain a sweat sample, under the guise that this would be used later to determine the participants objective state of sexual arousal during the session. This deception item was included to ensure participants completed the task as instructed (all aspects of the study were approved by Griffith University Human Research Ethics Committee; GU Ref No: 2014/577).

Participants were also asked to complete three short (i.e., to minimize inconvenience and maximize survey return) questionnaires at the end of their second session—these being included in the relevant study packet. These were: (1) The core disgust questions from the Disgust Sensitivity Questionnaire (Revised; Olatunji et al., 2008), (2) The Perceived Vulnerability to Disease questionnaire (PVD; Duncan et al., 2009), and (3) The Sociosexuality index (SOSI; Penke and Asendorpf, 2008).

### Procedure

Face-to-face contact between experimenter and participant was kept to a minimum to reduce embarrassment. Participants learned of the study via advertisements posted on social media, across the Griffith University campus, and on an undergraduate psychology research participation website. Each advertisement directed interested individuals to a website where more information regarding the study and its requirements were presented. This page provided straightforward information regarding the purpose of the experiment, the tasks to be performed by participants, as well as information regarding the compensation individuals would receive in return for their participation. Individuals who remained interested were directed to contact the study email address. Email correspondence between the participants and experimenters was standardized. A final information sheet was provided to individuals who emailed the researcher, and upon indicating their continued interest in the study, the participant could elect to collect the study materials from the Griffith University, School of Applied Psychology—Gold Coast reception or have the materials posted to their address.

Participants received two separate study packets to complete at home. Participants were instructed in the information sheet to abide by a minimum 24-h period between the completion of the first and second packet, with approximately half of them instructed to follow a neutral-arousal study packet order and vice versa. In the neutral study packet, participants were directed to complete the entirety of the study packet whilst in a “unaroused/neutral state.” In the self-arousal study packet, participants were instructed to “self-stimulate (masturbate) to a point of high but sub-orgasmic level of arousal (i.e., not to the point of ejaculation), and to then complete the rating tasks whilst remaining in this aroused state.” During the self-arousal session, participants were directed to take a sample of their forehead sweat using the provided gauze pad and were asked to place this sample in the bag provided. Participants were informed on the information sheet that perspiration can provide a reliable measure of sexual arousal and the collection of the sweat sample was to ensure that participants were completing the experimental task as instructed. This was the only form of deception used in the study and was employed to encourage participants to complete the task as instructed (i.e., in a high state of sexual arousal). Upon completion of both study packets, participants could elect to return them to the school reception or via mail. When participants presented to collect their payment for participation, they were provided with a debrief form which contained information regarding the use of deception in the study and were also given the choice to withdraw from participation without penalty.

### Analysis

Participants completed a manipulation check at the start and end of each session, so that we could ensure that their level of self-reported sexual arousal was: (1) higher at the start of the self-arousal session than at the start of the control session, and (2) that in the sexual arousal session all ratings exceeded 1 (i.e., above a report of “no sexual arousal”). Two participants reported being as aroused at the start of the non-arousal session as they were on the self-arousal session, so their data were excluded from the analysis. A further participant reported no sexual arousal at the end of the self-arousal session and was thus also excluded.

Participants were also asked about their sexual orientation, with one participant reporting being exclusively homosexual. This participant was also excluded from the study.

All of the data were suitable for parametric analysis (i.e., data were normally distributed [evaluated using skewness and kurtosis values], there was homogeneity of variance [established using Levene's test for between variables] and there were no violations of sphericity [established using Mauchly's test]). Due to the nature of the study several participants had missing data, which meant their exclusion from certain ANOVAs. For the regression analyses, we were able to impute some of the missing data based on averaging the remaining responses (e.g., for comparisons of the self-aroused vs. unaroused sessions “Willingness to have sex” ratings, at an individual level this was computed by subtracting the mean of the four “Willingness to have sex” ratings for each cell of the design [attractive vs. unattractive by sex-worker by non-sex worker] in the unaroused state, from the mean of those same four ratings in the self-aroused state. If one rating were missing, say the attractive sex-worker in the unaroused state, then the three remaining data points were averaged and this formed the score to subtract from the corresponding mean score in the self-aroused state; such imputation was conducted for 8 cases). We note that utilizing the extant data with no imputation yields similar, albeit less powerful, results.

We started by examining the manipulation check arousal ratings using a three-way mixed design ANOVA, with Arousal (self-arousal session vs. non-arousal session) and Time (rating at the start vs. end of the session) as within-factors and Session order (arousal-non arousal vs. non-arousal-arousal) as the between factor. The ratings of the images obtained on each session were analyzed using four-way mixed design ANOVAs, with Arousal (self-arousal session vs. non-arousal session), Attractiveness (attractive vs. unattractive) and Sex-worker status (Sex-worker vs. Control) as within-factors and Session order (arousal-non arousal vs. non-arousal-arousal) as the between factor. The final set of analyses examined predictors of the observed alterations in arousal and willingness for sex, that were identified in the analysis of the image ratings. Here we used a two-stage regression procedure, first entering state disgust and STI risk ratings of the images, and then, second, entering the more trait-based predictors, namely Core disgust sensitivity, Perceived vulnerability to disease and the Sociosexuality index. A data summary is available in Supplementary Material.

## Results

### Participants

The final sample consisted of 47 men, aged between 18 and 45 (M age = 23.9, *SD* = 6.1). Forty-two of these men reported being heterosexual and five bisexual. Core disgust sensitivity (DS) scores ranged from 0.5 to 9.5 (M DS = 5.4, *SD* = 2.1), PVD total scores ranged from 1.9 to 5.3 (M PVD = 3.2, *SD* = 0.8), and Sociosexuality Index (SOI) scores ranged from 1.9 to 8.9 (M SOI = 5.3, *SD* = 1.6)—thus in all cases providing a good degree of variability. There were no differences between these variables by the order in which the sessions were completed.

### Manipulation Check

Participants rated their degree of sexual arousal at the start and end of each session. The ANOVA revealed a main effect of Arousal, *F*_(1, 44)_ = 433.73, *p* = 0.0000152, η^2^ = 0.91, with this being qualified by an Arousal by Time interaction, *F*_(1, 44)_ = 18.63, *p* = 0.0000887, η^2^ = 0.30. On the control non-arousal session, sexual arousal increased from a starting mean of 1.4/7 (*SD* = 0.5) to 1.9/7 (*SD* = 1.0), while in the self-arousal session it waned from a starting mean of 5.8/7 (*SD* = 1.1) to 5.5/7 at the end of the session. Session order also exerted an effect. Sexual arousal was reportedly higher in participants who received the non-arousal session first (*M* = 3.9/7; *SD* = 0.7), relative to those who received the arousal session first (*M* = 3.5/7; *SD* = 0.8). In addition, there was a Session order by Time interaction, *F*_(1, 44)_ = 4.32, *p* = 0.04, η^2^ = 0.09, with a small reduction in arousal across Time in participants who received the self-arousal session first (*M diff* = 0.1; *SD* = 1.0), relative to those who received it second (*M diff* = 0.3; *SD* = 1.1). Irrespective of these differences, it is readily apparent that participants reported considerably greater sexual arousal in the self-arousal session than in the control non-arousal session, both at its start and finish.

### Analysis of the Image Ratings

Table 1 presents the mean values (and standard deviations) for all of the dependent and independent variables used in the study. Table 2 details the ANOVA results from the analyses.

**Table 1:** Means (and standard deviations) for each dependent variable organized by the experimental manipulations.

**Independent variables**	**Dependent variables**			
	**Disgust**	**STI risk**	**Arousal**	**Willingness for sex**
	***M* (*SD*)**	***M* (*SD*)**	***M* (*SD*)**	***M* (*SD*)**
**UNAROUSED STATE**				
Attractive control	1.7 (1.3)	2.5 (1.1)	3.2 (1.4)	4.0 (1.7)
Attractive sex-worker	3.9 (2.0)	5.2 (1.4)	2.7 (1.6)	2.5 (1.4)
Unattractive control	2.2 (1.4)	2.5 (1.2)	3.0 (1.4)	3.6 (1.4)
Unattractive sex-worker	4.2 (2.0)	5.2 (1.4)	2.4 (1.4)	2.6 (1.8)
**AROUSED STATE**				
Attractive control	1.5 (1.1)	2.3 (1.2)	4.8 (1.3)	5.2 (1.3)
Attractive sex-worker	3.7 (1.9)	4.7 (1.7)	4.2 (1.8)	4.4 (1.9)
Unattractive control	1.8 (1.3)	2.1 (1.1)	4.1 (1.5)	5.0 (1.3)
Unattractive sex-worker	3.6 (2.1)	4.8 (1.6)	3.5 (1.8)	4.0 (2.2)

**Table 2:** Analysis of variance results for each dependent variable organized by main effects and interactions.

**Effects**	**Dependent variables**			
	**Disgust *F*_**(1, 41)**_ =**	**STI risk *F*_**(1, 42)**_ =**	**Arousal *F*_**(1, 42)**_ =**	**Willingness for sex *F*_**(1, 40)**_ =**
Arousal (AR)	4.94, *p* = 0.03, η^2^ = 0.11	8.21, *p* = 0.006, η^2^ = 0.16	110.46, *p* = 1.26 × 10^−5^, η^2^ = 0.73	70.32, *p* = 1.26 × 10^−5^, η^2^ = 0.64
Attractive (AT)	6.26, *p* = 0.02, η^2^ = 0.13	< 1	9.51, *p* = 0.004, η^2^ = 0.19	< 1
Sex-work status (SW)	83.04, *p* = 1.26 × 10^−5^, η^2^ = 0.67	141.68, *p* = 1.26 × 10^−5^, η^2^ = 0.77	4.86, *p* = 0.03, η^2^ = 0.10	17.78, *p* = 0.0001, η^2^ = 0.31
Session order (SO)	< 1	4.60, *p* = 0.04, η^2^ = 0.10	< 3.9	< 1
AR × AT	< 1.5	< 1	7.44, *p* = 0.09, η^2^ = 0.15	< 1
AR × SW	< 1.5	< 1	< 1	5.08, *p* = 0.03, η^2^ = 0.11
AR × SO	< 1	8.21, *p* = 0.006, η^2^ = 0.16	11.98, *p* = 0.001, η^2^ = 0.22	4.40, *p* = 0.04, η^2^ = 0.10
AT × SW	< 1	< 1	< 1	< 1
AT × SO	< 1	< 1	< 1	< 1
SW × SO	< 1	< 3.5	6.91, *p* = 0.01, η^2^ = 0.14	< 1
AR × SW × SO	< 1	< 1	< 2.1	< 2.7
AT × AR × SO	< 1	< 1	< 1	< 1
AT × AR × SW	< 1	< 1	< 1	< 2.1
AT × SW × SO	< 1	< 1	< 1	4.27, *p* = 0.05, η^2^ = 0.10
AT × AR × SW × SO	< 1.5	< 1	< 1.3	< 1

For disgust ratings, all three main effects were significant, but with no interactions or session order effects (see Table 2). Consistent with our first hypothesis, greater sexual arousal was associated with a mean reduction in disgust of 4.7%. Unattractive images were judged a mean of 5.0% more disgusting than attractive images. Images of sex-workers were judged a mean of 30.1% more disgusting than controls. Notably, all of these effects were independent, such that when disgust fell following sexual arousal, this reduction was evident irrespective of whether the image being rated was attractive or unattractive, and of a sex-worker or a control.

Disease risk ratings mirrored disgust ratings for the main effects of Arousal and Sex-worker status, but Attractiveness had no impact on this type of evaluation (see Table 2). Sex-worker status exhibited the largest effect on risk ratings, with sex-workers judged a higher disease risk than controls by a mean of 36.8%. Consistent with our first hypothesis, sexual arousal was associated with a mean fall of 5.6% in disease risk ratings. However, the order in which the sessions were completed significantly affected this. Participants, who completed the self-arousal session first, reported the same level of disease risk on both sessions (M self-arousal session = 3.9/7, *SD* = 1.1; M non-aroused session = 3.9/7, *SD* = 1.3), while participants who completed the non-arousal session first reported reduced disease risk on their arousal session (M self-arousal session = 3.0/7, *SD* = 1.4; M non-aroused session = 3.8/7, *SD* = 1.2). This would suggest that sexual-arousal only reduces risk assessment when there is non-arousing pre-exposure to the stimuli—an unexpected outcome. There was also a main effect of Session order, but this was of lesser interest due to the interaction.

Participants also evaluated their willingness to have sex with the people depicted in the images (see Table 2). Participants reported a mean 20.7% greater willingness to have sex following the arousal manipulation. Sex-worker status affected willingness to have sex, with a mean reduction of 15.1% when the depicted person was a sex-worker. There was an interaction between the Arousal manipulation and Sex-worker status. There was a greater mean increase in willingness to have sex with a sex-worker (*M* = 23.6%), relative to a control (*M* = 17.7%), when contrasting the self-arousal session to the control session. There were also two effects involving order. First, participants who completed the self-arousal session first, reported a smaller increase in willingness to have sex across sessions (*M diff* = 1.1, *SD* = 1.6), than participants who completed the non-arousal session (*M diff* = 1.9, *SD* = 1.8). Second, participants who had completed the self-arousal session first, reported a smaller difference in willingness to have sex between appealing control and sex-workers (*M diff* = 0.9, *SD* = 2.0), when contrasted to unappealing control and sex workers (*M diff* = 1.3). This relationship was reversed for participants who completed the self-arousal condition second (corresponding values; *M diff* = 1.4, *SD* = 2.3; *M diff* = 0.7, *SD* = 1.9).

Finally, participants judged how sexually arousing they found each type of image. Four effects were evident here (see Table 2). All three main effects were significant, and in addition Attractiveness interacted with Arousal. In the self-arousal session, participants judged all of the images a mean of 19.1% more arousing than when they viewed them in the control session. However, this effect interacted with Session order. Participants who completed the self-arousal session first, demonstrated a smaller difference in arousal between the two sessions (*M diff* = 0.9, *SD* = 1.3), than participants who completed the control session first (*M diff* = 1.8, *SD* = 1.4). Images of sex-workers were judged a mean 7.5% less sexually arousing than images of controls and this too interacted with Stimulus order. Here, participants who completed the arousal session first reported a much larger drop in arousal between the images of the control and sex-worker pictures (*M diff* = −1.1, *SD* = 1.0), than participants who completed the control condition first (*M diff* = 0.0, *SD* = 1.6). Attractive images were rated a mean 7.0% more sexually arousing than unattractive images. Attractiveness and Arousal interacted, with attractive images demonstrating a much larger change in their capacity to arouse across the self-arousal and control sessions (*M* = 22.1%), than unattractive images (*M* = 16.2%).

### Predictors of Change

Judgments of how sexually arousing the images were and how willing the participant was to have sex them, were both significantly affected by the self-arousal manipulation and by whether the image was of a sex-worker or control. Here we examined whether individual variation in these effects could be predicted by disgust and disease risk measures obtained in the study, and by trait measures of core disgust sensitivity, perceived vulnerability to disease and attitudes to short-term relationships. We note that none of the effects examined here, differed by Session order, even when tested (Williams test) without correction for multiple comparisons.

As we predicted in our second hypothesis, reductions in disease risk judgments were positively associated with reductions in disgust (see Table 3). Across arousal state, judgments of both willingness to have sex and of how arousing the images were, increased in unison (see Table 3). Consistent with hypothesis 3(1), both of these increases were correlated with decreases in risk judgments. However, these increases were not related to changes in disgust, refuting the other part of hypothesis 3(1). For the trait measures, greater core disgust sensitivity was associated with larger changes in arousal, with a similar trend for willingness. These findings were largely borne out by the regression analyses detailed in Table 4. Larger changes in ratings of willingness for sex and of arousal, were most reliably predicted by reductions in STI risk rating, higher trait disgust and lower perceived vulnerability to disease. The differing outcomes for state and trait disgust were consistent with hypothesis 4.

**Table 3:** Pearson correlations between rating difference scores for change in arousal state and trait scores.

**Variable**	**Change in arousal state (self-arousal session—control)**				**Trait scores**	
	**Δ Willingness**	**Δ Arousal**	**Δ Risk**	**Δ Disgust**	Core DSQ^*^	PVD total^*^
ΔArousal	0.69^†^					
ΔRisk	−0.50^†^	−0.43^†^				
ΔDisgust	−0.18	−0.18	0.50^†^			
Core DSQ^*^	0.27	0.33^†^	−0.03	−0.13		
PVD total^*^	−0.06	−0.17	−0.17	0.04	0.27	
SOSI^*^	0.15	0.13	−0.13	−0.24	0.01	0.06

* *Core DSQ, Disgust Sensitivity Questionnaire Revised; PVD total, Perceived vulnerability to disease; SOSI, Socio-sexuality index*.

† *p < 0.05*.

**Table 4:** Regression analyses.

**Model and predictors**	**Change in arousal state (self-arousal session-control)**		**Change in sex-worker status (control—sex-worker)**	
	**Willingness for sex rating**	**Sexual arousal rating**	**Willingness for sex rating**	**Sexual arousal rating**
Model 1	*F*_(2, 41)_ = 7.89, *R*^2^ = 0.24^*^	*F*_(2, 39)_ = 4.29, *R*^2^ = 0.14^*^	*F*_(2, 44)_ = 1.86, NS	*F*_(2, 44)_ = 0.20, NS
STI risk	β = −0.57, *p* = 0.001	β = −0.45, *p* = 0.011	β = 0.27, NS	β = 0.08, NS
Disgust	β = 0.10, NS	β = 0.05, NS	β = −0.13, NS	β = 0.05, NS
Model 2	*F*_(5, 38)_ = 5.34, *R*^2^ = 0.34^*^	*F*_(5, 36)_ = 5.92, *R*^2^ = 0.38^*^	*F*_(5, 41)_ = 2.59, *R*^2^ = 0.15^*^	*F*_(5, 41)_ = 0.75, NS
Fit improvement	*F*_(3, 38)_ = 2.90, *p* = 0.048	*F*_(3, 36)_ = 5.93, *p* = 0.002	*F*_(3, 41)_ = 2.91, *p* = 0.046	*F*_(3, 41)_ = 1.11, NS
STI risk	β = −0.63, *p* = 0.0001	β = −0.57, *p* = 0.001	β = 0.23, NS	β = 0.04, NS
Disgust	β = 0.21, NS	β = 0.23, NS	β = −0.18, NS	β = 0.00, NS
Core DS^**^	β = 0.33, *p* = 0.02	β = 0.43, *p* = 0.002	β = −0.36, *p* = 0.016	β = −0.26, NS
PVD^**^	β = −0.27, *p* = 0.056	β = −0.44, *p* = 0.002	β = 0.30, *p* = 0.041	β = −0.05, NS
SOSI^**^	β = 0.12, NS	β = 0.14, NS	β = 0.05, NS	β = 0.00, NS

* *p < 0.05, R values are adjusted*.

** *Core DS, Disgust Sensitivity Questionnaire Revised; PVD, Perceived vulnerability to disease total; SOSI, Socio-sexuality index total*.

We also examined changes in reported willingness to have sex, and how arousing the images were, when contrasting controls with sex-workers. The correlations, reported in Table 5, indicate that changes in arousal and willingness ratings were again related, with a trend for both of these variables to be associated with trait core disgust—nothing else was evident, refuting hypothesis 3(2). The regression analyses again reflected these findings (see Table 4). For willingness to sex, the final model was significant, with core disgust sensitivity and perceived vulnerability to disease the only significant predictors. Lower disgust sensitivity and lower perceived vulnerability to disease was associated with a larger difference in reported willingness to have sex between a control and a sex-worker. For the sexual arousal rating regression, the models were not significant. The differing outcomes for state and trait disgust were consistent with hypothesis 4.

**Table 5:** Pearson correlations between rating difference scores for change in sex-worker status and trait scores.

**Variable**	**Change in Sex-worker status (control—sex-worker)**				**Trait scores**	
	**Δ Willingness**	**Δ Arousal**	**Δ Risk**	**Δ Disgust**	Core DSQ^*^	PVD total^*^
Δ Arousal	0.38^†^					
Δ Risk	0.25	0.08				
Δ Disgust	−0.10	0.06	0.14			
Core DSQ^*^	−0.28^††^	−0.28^††^	−0.16	−0.19		
PVD total^*^	0.21	−0.12	−0.04	−0.07	0.27	
SOSI^*^	0.03	0.00	−0.01	0.15	0.01	0.06

* *Core DSQ, Disgust Sensitivity Questionnaire Revised; PVD total, Perceived vulnerability to disease; SOSI, Socio-sexuality index*.

† *p < 0.05*,

†† *p = 0.055*.

We then conducted a second set of regression analyses on the remaining significant effects for the willingness to have sex and arousal ratings reported in Table 2 (i.e., Attractiveness, Arousal by Attractiveness for arousal ratings; Arousal by Sex-worker status for willingness ratings). There were no significant effects (refuting hypothesis 3(3)), when using the same set of predictor variables as detailed in the preceding analyses.

## Discussion

This study examined the role of disgust in STI-related sexual decision-making in young men. Consistent with prior findings of reduced object-related disgust with higher sexual arousal (Stevenson et al., 2011; Borg and de Jong, 2012), and with our first hypothesis, we found that disgust judgments of potential sexual partners were reduced when participants were sexually aroused. Similarly, and consistent with previous studies (Blanton and Gerrard, 1997; Shuper and Fisher, 2008; Imhoff and Schmidt, 2014; Skakoon-Sparling et al., 2016) and with our first hypothesis, judgments of STI risk were also reduced, and as predicted in our second hypothesis, this reduction in risk was significantly associated with the fall in disgust. Participants also judged how sexually arousing the target images were and how willing they were to have sex with the person depicted in them. These ratings increased when participants were aroused and when they depicted images of control women. Attractiveness had little effect on willingness for sex, but a greater effect on arousal ratings. Overall, there were few interaction effects. We also explored whether disgust and STI risk ratings, along with trait-related measures, could predict participants arousal and willingness for sex ratings. In partial support of our third hypothesis (first part), when participants were sexually aroused, in contrast to when they were unaroused, disease risk ratings but not disgust ratings, were significantly predictive of changes in willingness for sex with, and arousal for, all of the depicted images. Trait disgust and perceived vulnerability to disease were also found to be significant predictors, but these effects, as we suspected (hypothesis four) did not parallel trait disgust measures. Finally, for judgments most directly connected with STI risk perception—between images depicting sex-workers vs. controls, only trait disgust and perceived vulnerability to disease were found to be predictive—contrary to the second part of hypothesis three.

Before turning to the implications of these findings, it is important to reflect upon their validity. First, while we could not take any physiological measures of arousal due to the way that we designed the study (i.e., conduct at home), we note that self-report measures of sexual arousal in men have been found to closely correspond to objective measures of arousal in several studies (see Chivers et al., 2010). Second, as we noted above, our findings follow expectations derived from prior laboratory studies, including reductions in disgust and STI risk perception under conditions of sexual arousal, suggesting convergent validity. Third, participants—as many did—could opt out of the study simply by failing to return the study packets. This can be taken to imply that those who did return the study packets, did so having followed the study instructions, and in the knowledge that their sweat sample would be tested for evidence of sexual arousal. While we cannot be sure that participants reported sexual intentions would actually translate into behavior—an issue for all studies in this area—it would seem likely that our participants did as they were asked to do. Fourth, participants self-selected for inclusion in this study, and so it is possible that as with all other studies in this area, they may be unrepresentative of the broader young male population. Nonetheless, their scores on the self-report individual differences measures did not suggest that the sample were particularly unusual. Finally, we also note that these findings may not generalize to the entire male population, although some of our results replicate effects observed in different samples of men and women.

As predicted sexual arousal (in comparison to a non-aroused state), increased ratings of willingness to have sex, and judgments of how arousing the target images were. This change in willingness and image arousal ratings was significantly predicted by the change in risk perception, but not by change in disgust ratings (i.e., state). In addition, we also found that trait disgust and perceived vulnerability to disease explained additional variance here. Ideally, these relationships need to be examined using structural equation modeling—something precluded here because of our sample size and due to the exploratory nature of this study—nonetheless our data suggest a plausible model for future testing. We suggest that state disgust, namely that felt at a particular moment, contributes to a risk decision, presumably alongside other inputs such as a person's knowledge of STI transmission. This combined risk measure then informs both arousal and willingness judgments. In addition, there is a second and seemingly independent influence of disgust, through trait core disgust sensitivity. Here, greater trait core disgust sensitivity is associated with a greater propensity for sexual arousal to drive changes in willingness to have sex and image arousal ratings. What this may mean is that individuals who are generally highly disgust sensitive, are those who most strongly change behavioral intentions under conditions of sexual arousal. We note here the interesting parallel with Grauvogl et al. (2015), who found that high levels of trait disgust were linked to greater genital and self-reported sexual arousal. Thus, we suggest that state disgust has an indirect effect on risk perception, which in turn affects willingness and arousal ratings, while trait core disgust has a direct effect on both.

We also examined the predictors of differences in willingness for sex and arousal ratings for images depicting sex-workers and age-matched controls. While there was a close yoking between state disgust and willingness (and arousal ratings)-−88% of participants reported greater disgust for sex-workers *and* a reduced willingness for sex, these two variables were not significantly associated. However, trait disgust, and perceived vulnerability to disease, were both correlated with these difference scores. Individuals who were high in core disgust sensitivity reported a much smaller difference in willingness for sex (with a similar albeit non-significant trend for arousal) between images of sex-workers and controls. In this case, we suggest that trait core disgust reflects a high baseline level of responding to the possibility of sex with all of the images, thereby reducing the magnitude of the difference between willingness and arousal ratings for sex-workers and controls. Perceived vulnerability disease (PVD) had a different relationship. A greater PVD score was linked to a greater difference in response between willingness ratings for sex-workers and controls. That is being germ averse and wary of disease reduced willingness for sex, both here, and similarly so, for changes in sexual arousal.

We did not find an effect of attractiveness on willingness for sex, although it was apparent for the image arousal ratings. What we did find though, was that unattractive images were judged as significantly more disgusting, independent of other effects. This is an interesting observation, because it points again to the possible evolutionary basis of disgust as a disease avoidance system. Attractiveness, in all of its facets, is generally thought to reflect various aspects of health, and thus a key aspect of potential mate value (Buss, 2015). To the extent that disgust is driven by cues that are indicative of disease—directly and indirectly (i.e., indicators of poor health)—unattractiveness might then be regarded as one such indirect cue.

We also found unexpected effects of order. Specifically, participants that first viewed and rated the study stimuli in an unaroused state, and then in an aroused state, reported a greater reduction in perceptions of disease risk (about the target) and a greater willingness for sex (with the target), relative to participants that viewed and rated the study stimuli in the opposite arousal order. Whilst these findings were not predicted, they are not surprising. Prior research on sexual risk taking has shown that increased familiarity with a potential sexual target encourages appraisals that the target is low in disease risk (Swann et al., 1995). Swann et al. (1995) reported that receiving 1 min of video-taped information about a potential sexual target, even though the information was irrelevant to the target's sexual health status, increased participants' feelings of familiarity and liking, and decreased appraisals of disease risk. Potential sexual partners who are familiar may be perceived as safe due to person perception biases and the reliance on incorrect heuristics to estimate a potential sexual partner's disease risk. For example, familiarity may influence disease risk perceptions via social projection bias, which is the tendency to expect similarities between ourselves and others, especially those who are familiar to us (Robbins and Krueger, 2005). Most young adults consider themselves at low risk of STIs (Fromme et al., 1999), and may project this perception of self to familiar others. Our findings are tentative, but suggest that familiarity with a potential sexual partner can operate as a situational cue that the potential partner is low in sexual disease risk and therefore condom use, for example, is not warranted.

In conclusion, we have suggested that disgust may play multiple roles in STI-related sexual decision making, both at a state and trait level. Two important issues emerge for further study. First, we did not assess other potential domains of trait disgust, partly because there is some disagreement over exactly what these other domains might be—noting however that core disgust is widely agreed to exist (i.e., Olatunji et al., 2008; Rozin et al., 2016)—and also because we were restricted in how many questions we could ask participants to complete. Second, we were unable to complete structural equation modeling, as we did not have sufficient number of cases nor a clear enough *a priori* model from which to work. We suggest that the data reported here provides an empirical framework for such an approach. Finally, we note more broadly that these findings implicate disgust as a component of sexual decision-making in the context of STI risk.

## Author Contributions

MO, RS, and TC conceived of the study. MO and RS designed the study. MO, CT, and AC coordinated and administered the study. MO, RS, and CT participated in data and statistical analysis. All authors helped draft the manuscript, and gave final approval for publication.

### Conflict of Interest Statement

The authors declare that the research was conducted in the absence of any commercial or financial relationships that could be construed as a potential conflict of interest.

## Supplementary Material

Supplementary Data Sheet 1 Data Summary: The role of disgust in male sexual decision-making; Oaten et al. (2009).
